# Identification of *Leishmania* Species Using PCR Assay on Giemsa-Stained Slides Prepared From Cutaneous Leishmaniasis Patients

**Published:** 2013

**Authors:** Farnaz KHEIRANDISH, Ali CHEGENI SHARAFI, Bahram KAZEMI, Mehdi MOHEBALI, Amanollah SARLAK, Mohamad Javad TARAHI, Kourosh HOLAKOUEE, Homa HAJARAN

**Affiliations:** 1 Razi Herbal Medicines Research Center, Department of Parasitology and Mycology, Lorestan University of Medical Sciences, Khorramabad, Iran; 2 Department of Communicable Disease Control and Prevention, Deputy of Health, Lorestan University of Medical Sciences, Khorramabad, Iran; 3 Cellular and Molecular Biology Research Center, Shahid Beheshti University, M.C., Tehran, Iran; 4 Department of Parasitology and Mycology, School of Medicine, Shahid Beheshti University of Medical Sciences, Tehran, Iran; 5 Department of Biotechnology, School of Medicine, Shahid Beheshti University of Medical Sciences, Tehran, Iran; 6 Department of Parasitology & Mycology, School of Public Health, Tehran University of Medical Sciences, Tehran, Iran; 7 Department of Epidemiology, School of Public Health, Tehran University of Medical Sciences, Tehran, Iran

**Keywords:** *Leishmania*, Giemsa, PCR, Cutaneous leishmaniasis, Iran

## Abstract

**Background:**

Leishmaniasis is a group of diseases that are created by intracellular parasites of *Leishmania*. Cutaneous leishmaniasis is considered as one of the health problems in some provinces of Iran.

**Methods:**

In this study, a total of 178 Giemsa-stained slides from confirmed cases of cutaneous leishmaniasis were examined. The slides were prepared from the patients with cutaneous leishmaniasis that referred to health centers and infected during the epidemic of cutaneous leishmaniasis in Poldokhtar city, Lorestan Province, Iran in 2006.Genomic DNA from each slide was extracted. After DNA extraction, ITS-PCR was used.

**Results:**

Out of 178 slides, 129 (72.47%) samples had a band in the range of 485 bp and 49 (27.53%) samples 626 bp that matched *L. tropica* and *L. major* standard samples, respectively.

**Conclusion:**

This study showed that *Leishmania* DNA could be efficiently extracted and amplified even from old Giemsa-stained microscopic slides that were stored more than 6 yr. In this study was shown that both *L. tropica* and *L. major* species exist in Lorestan Province.

## Introduction

Leishmaniasis is a group of diseases that are created by intracellular parasites of *Leishmania*. Out of 30 species of the recognized *Leishmania*, about 10 species are important due to medical and veterinary (1). This disease is observed in three main forms of cutaneous, visceral and mucocutaneous leishmaniasis (2). Due to the importance of health in this disease, it has always been considered by the World Health Organization. Currently in 98 countries or territories leishmaniasis is endemic (3). Prevalence rate of leishmaniasis have been reported around 12 million cases (3–5).

Cutaneous leishmaniasis is considered as one of the health problems in some provinces of Iran and more than 20 thousand cases of this disease are reported annually (6). In Iran, *Leishmania L. major* is agent of zoonotic cutaneous leishmaniasis (ZCL), and *L. tropica* causes anthroponetic cutaneous leishmaniasis (ACL) (2, 7, 8).

Considering the biological properties of the parasite, reservoir and vector, the methods to combat these two forms of cutaneous leishmaniasis, are different. Therefore, identification of parasite species is important in the health planning (9).

For molecular studies, the different parts of *Leishmania* genome are used, one of them that has many applications is ribosomal gene used for ITS amplification fragment (10, 11). The advantages of these methods are that even with the low number of parasite, the infection is shown and parasite species identified (9, 10).

Cutaneous leishmaniasis is reported in several provinces of Iran and the species characterization in some of them was done by PCR method. For example, in endemic foci of south-eastern Iran was shown that *L. tropica* is the main species caused ACL and *L. major* is present in low level (12). A study in Qom Province were done by PCR and showed that *L. major* is the causative disease (13). In the studies on CL in Mashhad using ITS-PCR and PCR-RFLP, was determined that *L. tropica* was the dominant species (14, 15). Also in order to identify *Leishmania* species using Nested–PCR, the studies were performed in Shush city in Khuzestan Province and Shiraz city and reported that the predominant species was *L. major* (16, 17).

Lorestan Province is one of the endemic areas in Iran that both species of *L. tropica* and *L. major* have been reported in this province (18). Since the epidemic of cutaneous leishmaniasis happened in 2006 (19) and probable of its occurrence, to know which species are dominant in the epidemic is especially important.

This study was carried out for the first time to evaluate of PCR in order to detect of *Leishmania* DNA and molecular identification of *Leishmania* species in archived Giemsa-stained slides in Poldokhtar City, Iran.

## Materials and Methods

The study was performed in Parasitology Department, Cellular and Molecular Research Center, Lorestan University of Medical Sciences, Iran.

A total of 178 Giemsa-stained slides from confirmed cases of cutaneous leishmaniasis were examined. The slides were prepared from the patients with cutaneous leishmaniasis that referred to health centers and infected during the epidemic of cutaneous leishmaniasis in Poldokhtar City, Lorestan Province, Iran in 2006.

DNA from each slide was extracted separately by AccuPrep^®^ Genomic DNA Extraction Kit (Bioneer, South Korea) in accordance with the manufacturer's protocol. Extracted DNA was stored at - 20 °C until PCR amplification. The ribosomal internal transcribed spacer (ITS) was amplified with specific primers according to previous studies (8), LeishF (5‘- CAA CACGCCGCCTCCTCTCT -3‘), LeishR (5‘- CCTCTCTTTTTTCNCTGTGC-3‘). The PCR conditions consisted of one initial denaturing cycle at 94°C for 5 min, followed by 30 cycles of 94 °C for 30 s, 56 °C for 30 s, 72 °C for 40 s and finally 1 cycle of 72° C for 5 min (8). At the end, PCR products were analyzed by 1.5% agarose gel electrophoresis.

Expected PCR products for *L. major* and *L. tropica* were 626 bp, 485 bp, respectively. Standard strains of *L. major* ((MRHO/IR/75/ER) and *L. tropica* (MHOM/IR/01/yaza) were used as positive controls.

Negative controls were used. In one of them was added distilled water instead of DNA. In another one extracted DNA was used that it was prepared from cutaneous lesion slide of patient with skin disease except CL which *Leishmania* was not found in lesion using microscopic exams, culture and PCR.

### DNA sequencing

DNA for sequencing was prepared by the ITS-PCR.

## Results

**Fig. 1:**
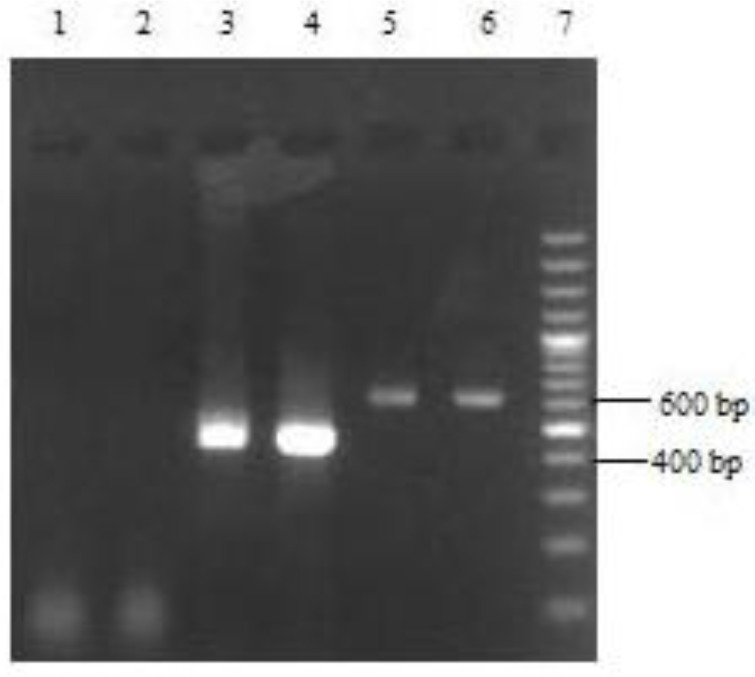
1.5% agarose gel electrophoresis of PCR amplification for identification of *Leishmania* species on Giemsa-Stained slides Lane 1, 2: Negative controls Lane 3: *L. major* isolate Lane 4: Reference strain of *L. major* Lane 5: *L. tropica* isolate Lane 2: Reference strain of *L. tropica* Lane 7: 100bp DNA ladder marker

Out of 178 slides, 111 (62.36%) of whom were for male and 67 (37.64%) for female and also 14 (7.87%) slides were collected from urban and 164 (92.13%) from rural. Frequency distribution of cutaneous leishmaniasis base on age, number and location of the lesion were shown in Table 1 and Table 2, respectively. Electrophoresis patterns from each isolates were compared with reference strains of *L. tropica* and *L. major*. After DNA extraction and PCR performance, according to the pattern of electrophoresis, out of 178 slides, 129 (72.47%) samples had a band in the range of 485 bp and 49 (27.53%) samples 626 bp that matched *L. tropica* and *L. major* standard samples, respectively (Fig. 1). Number of *Leishmania* amastigotes on Giemsa-stained smears was shown in Table 3.

**Table 1:** Frequency distribution of cutaneous leishmaniasis base on age in Poldokhtar City, Iran in 2006

Age group (yr)	0-9	10-19	20-29	30-39	40-49	≥ 50	Total
**Frequency**	26	46	37	29	19	21	178

**Table 2:** Frequency distribution of cutaneous leishmaniasis base on the number and location of the lesion in Poldokhtar City, Iran in 2006

Location	Number of the lesions			
	1	2	≥3	Total
**Face**	16	6	0	22
**Foot**	28	14	7	49
**Hand**	54	18	23	95
**Face & Hand**	0	5	3	8
**Face & Hand & Foot**	0	0	1	1
**Hand & Foot**	0	1	2	3
**Total**	98	44	36	178

**Table 3:** *Leishmania* amastigote numbers on Giemsa-Stained slides prepared from cutaneous leishmaniasis patients in Poldokhtar City, Iran in 2006

*Leishmania species*	*Number of Amastigotes				Total
	1+	2+	3+	4+	
*L. tropica*	25	31	46	27	**129**
*L. major*	11	28	4	6	**49**
Total	36	59	50	33	**178**

* Grading of *Leishmania* Parasites was obtained by average parasite density using x10 eyepiece and x100 oil-immersion lens as follows: 4+ 1-10 parasites/fields/3+ 1-10 parasites/10 fields/2+ 1-10 parasites/100 fields/1+ 1-10 parasite/1000 field

Nucleotide sequence data reported in this article have been submitted to the GenBank database with accession numbers, *L. tropica* JX183382 and *L. major* JX183383.

## Discussion

Identification of the epidemiological aspects of cutaneous leishmaniasis for control program is necessary. So it is especially imp-ortant to identify the parasite species, because different species may require distinct treatment regimens (20, 21).

Because all *Leishmania* species are very similar, identification of *Leishmania* species by microscopic examination of Giemsa-stained slides is not possible (22). In recent years molecular methods including PCR have been used for diagnosis of leishmaniasis (9, 10, 23–26). In endemic areas where more than one *Leishmania* species is present, diagnostic tools are required for detection and identification of parasites directly in samples (22).

ITS-PCR was used for diagnosis and characterization of *Leishmania* species on Giemsa-stained slides of the patients with cutaneous leishmaniasis referred to health centers that infected during the epidemic of cutaneous leishmaniasis in Poldokhtar City, Lorestan Province, Iran.

Similar studies were conducted in different parts of Iran and other countries for the diagnosis of cutaneous leishmaniasis by PCR Giemsa-stained slides. For example, in a study DNA was isolated from 92 Giemsa-stained smears that had been stored for up to 4 years and used for PCR-based diagnosis of *Leishmania* infection and demonstrated this method is effective (27). In another study, PCR-RFLP was conducted on 48 Giemsa-stained slides and reported that technique is an effective method (28). In a study using PCR was identified that archived Giemsa-stained slides were positive for CL which caused by *L. tropica* (29). A study in Brazil examined the ability of PCR to amplify *Leishmania* DNA from Giemsa-stained slides that prepared from American cutaneous leishmaniasis patients. The slides were stored for up to 36 years. The results showed that archived Giemsa-stained slides are a useful source of *Leishmania* DNA for performing clinical and epidemiological studies of leishmaniasis (30). A study on 102 Giemsa-stained slides by real-time PCR, sensitivity of this method was reported 98%. Archived slides that were stored more than 3 years can be use for *Leishmania* DNA extraction and amplification by real-time PCR (31). Another study was conducted to evaluate sensitivity and specificity of PCR in order to detect of *Leishmania* DNA in archived Giemsa-stained bone marrow slides for diagnosis of visceral leishmaniasis. The results showed this method is a suitable tool for confirming diagnosis in Kalaazar patients and useful in the diagnosis of complicated cases. Additionally, was announced Giemsa-stained slides are easily stored, do not require special storage conditions, can be easily posted to centers where PCR is available and making a super option for diagnosis in the field (32). The study was done on Giemsa-stained smears for Palestinian patients. DNA was extracted from each slide and subjected to PCR. PCR showed 87% sensitivity and 100% specificity. This study reported that Giemsa-stained slides are an easily usable sampling method for PCR (33). Our study similar to other studies was shown that PCR Giemsa-stained slide is effective. Identification of *Leishmania* species using ITS-PCR technique on Giemsa-stained slides is an accurate and useful method that can be used in other endemic areas of leishmaniasis in Iran. Also in this method, there is no need to parasite cultivation or injection into laboratory sensitive animals and identification of *Leishmania* species is possible directly by the slides stained. Additionally, Giemsa-stained slides are appropriate for field condition such as samples can be easily stored and sent to the diagnostic laboratory (22, 27, 32) and can be helpful when re-evaluating the diagnosis of controversial cases or in retrospective epidemiologic studies (22).

This study like previous studies showed that *Leishmania* DNA could be efficiently extracted and amplified even from old Giemsa-stained microscopic slides those were stored more than 6 years.

Since in 2005, only 9 cases of cutaneous leishmaniasis were reported in Poldokhtar City, and in 2006, 178 cases that is a sign of epidemic of this disease in this year (19).

According to our results, out of 178 slides examined, 129 (72.47%) slides were *L. tropica* and 49 (27.53%) *L. major*. In previous study that was done on identification of *Leishmania* species of 43 patients with cutaneous leishmaniasis in Poldokhtar City, the results showed that out of 43 patients, 6 (13.95%) were infected with *L. major* and 37 (86.05%) with *L. tropica* (18). To compare these two studies shows that there is a significant difference between the prevalence of each species in the two studies so that during the epidemic, *L. major* frequency had been more than expected due to its prevalence in the region (*P*<0.05).

One of the reasons of this epidemic could be the return of Lorestan troops from operational area of Ilam Province, one of the endemic area of cutaneous leishmaniasis in Iran, who deployed in silos around the city along with their belongings probably brought the *Leishmania*-infected vectors (19). Since the vectors of this parasite are found in abundance in the province so easily caused the spread of disease. Another likely reason could be an increase in human– vector contact. This is attributed to the development of villages and the spread of the human population into the habitats of the local vectors (34).

In this study, the disease was observed in all age groups but most cases were seen ranged in age from 10 to 40 year that could be due to more working these people outside of the home and the possibility of more contact with sandfly. In this study as previous study (18) was shown that both *L. tropica* and *L. major* species exist in the province. Since the reservoir of *L. major* is rodents, so their identification for the purpose of planning of disease control, especially in epidemics is important (35–37). As mentioned above, considering the biological properties of the parasite, reservoir and vector, methods of combating these two species are different, so an accurate and comprehensive planning in this regard should be designed (2).

## Conclusion

The PCR procedure is a suitable tool for direct diagnosis and identification of *Leishmania* species from Giemsa-stained slides. Also this method is useful for retrospective epidemiologic studies.
